# Dietary Administration of a Soybean Fermented Preparation Reshapes Gut Microbial Community Structure and Colonic Mucosal Features in BALB/c Mice

**DOI:** 10.3390/microorganisms14030524

**Published:** 2026-02-24

**Authors:** Hyeokjin Kwon, Jang won Seo, Myeongguk Jeong, Yeeun Kim, Chulhun L. Chang, Ji-ho Kim, Go-Eun Choi

**Affiliations:** 1 Department of Clinical Laboratory Science, College of Health Sciences, Catholic University of Pusan, Busan 46252, Republic of Korea; ghy8627@gmail.com (H.K.); audrnr04@gmail.com (M.J.); 2 Next-Generation Industrial Field-Based Specialist Program for Molecular Diagnostics, Brain Busan 21 Plus Project, Graduate School, Catholic University of Pusan, Busan 46252, Republic of Korea; 3 Department of Alternative Medicine, Kyonggi University, Suwon 16227, Republic of Korea; ilgob1@kyonggi.ac.kr; 4 Department of Translational Biomedical Sciences, Graduate School of Dong-A University, Busan 49201, Republic of Korea; yeeun0509@naver.com; 5 Department of Laboratory Medicine, School of Medicine, Pusan National University, Yangsan 50612, Republic of Korea; cchl@pusan.ac.kr; 6 Department of Laboratory Medicine, Pusan National University Yangsan Hospital, Yangsan 50612, Republic of Korea; 7 Department of Korean Medicine, Mediram Hospital, Seoul 04038, Republic of Korea; puissans@naver.com

**Keywords:** gut microbiota, rRNA operon sequencing, intestinal homeostasis, soybean fermented preparation, Multimune

## Abstract

Background/Aim: Fermented soybean-based products are known to influence gut microbial composition; however, the long-term effects of multicomponent soybean fermented preparations on gut microbiota and colonic mucosal features remain insufficiently characterized. This study examined the effects of a commercially available soybean fermented preparation (SFP), containing additional fermented plant and marine derived components, on gut microbial community structure and colonic histological features in BALB/c mice. Methods: BALB/c mice received oral SFP (1000 mg/kg) for 30 and 60 days. Gut microbial communities were analyzed using full-length rRNA operon sequencing. Colonic mucosal architecture and goblet cell density were evaluated via histological analysis (H&E). Results: SFP supplementation induced significant β-diversity separation at both 30 and 60 days (*p* < 0.05), indicating consistent restructuring of the gut microbial community. While alpha diversity (Observed OTUs) remained stable at 30 days, Shannon and Simpson indices were significantly reduced at 60 days (*p* = 0.001), indicating reduced community evenness driven by increased dominance of specific taxa, including *Duncaniella*. At the genus level, SFP administration was associated with increased relative abundances of *Akkermansia*, *Lactobacillus*, and *Duncaniella*, accompanied by reductions in several genera previously linked to dysbiosis. Histological analysis demonstrated a significant increase in goblet cell density (*p* < 0.01) in SFP-treated mice. Conclusions: Long-term SFP supplementation was associated with sustained alterations in gut microbial composition and measurable histological changes in the colonic mucosa. While these findings indicate that SFP intake influences microbial structure and goblet cell abundance, further studies are required to determine the functional and physiological implications of these changes, particularly in relation to epithelial barrier function and host health.

## 1. Introduction

The human gastrointestinal tract harbors over 1000 species of bacteria, comprising more than 10^14^ individual microorganisms. These microorganisms play crucial roles in the host’s metabolism, immune regulation, and neuroendocrine functions. The gut microbial community contributes to energy balance by converting dietary components into various metabolic products, enhances intestinal epithelial barrier function, and suppresses the invasion of pathogenic microorganisms. Furthermore, specific members of the gut microbiota regulate both innate and adaptive immunity and mitigate systemic inflammatory responses. Consequently, the balance of the gut microbial ecosystem is closely linked to human health and disease prevention [1,2,3].

Modern dietary patterns characterized by high fat and refined carbohydrate intake have been shown to disrupt gut microbial homeostasis, leading to increased Firmicutes to Bacteroidetes (F/B) ratios, enhanced energy extraction, and chronic low-grade metabolic inflammation. Such dysbiotic shifts promote epithelial barrier impairment and immune activation, contributing to metabolic dysfunction. Several taxa, including *Oscillibacter*, *Alistipes*, and *Kineothrix*, are consistently enriched in high fat diet models and have been associated with obesity related inflammatory states and reduced mucosal resilience [4,5,6,7,8]. These observations highlight the importance of dietary interventions capable of restoring microbial balance and mitigating inflammation-driven dysbiosis [9].

Although changes in the F/B ratio and the abundance of so-called beneficial or pathogenic taxa are often discussed as indicators of gut health, growing evidence suggests that these measures are highly context-dependent and should not be interpreted as universal biomarkers. The F/B ratio can vary according to host genetics, dietary composition, and experimental conditions, and individual taxa may exert different effects depending on the physiological context. Therefore, gut microbiota alterations should be interpreted with caution, with greater emphasis on overall community structure rather than isolated taxonomic labels.

Beneficial bacteria including *Lactobacillus*, *Akkermansia muciniphila*, and *Alistipes* support epithelial barrier integrity, regulate inflammatory responses, and contribute to metabolic stability. For example, *A. muciniphila* degrades mucin in the intestinal mucosal layer and has been associated with improved metabolic health markers in preclinical studies [7,10,11]. In contrast, pathogenic species such as *Clostridioides difficile* and enterotoxigenic *Escherichia coli* induce inflammation, damage the epithelial lining, and disrupt immune function. Thus, the composition and diversity of the gut microbiota act as critical determinants of host immune and metabolic health [12,13,14].

In light of this, fermented foods have gained increasing attention for their potential to beneficially modulate the gut microbial environment [15,16]. Among fermented foods, soybean derived products contain high levels of isoflavones, saponins, and polyphenols, which undergo bioconversion during fermentation, leading to improved physiological activity and potentially favorable effects on the gut environment [17,18].

The fermentation of soybeans promotes the release and microbial conversion of tryptophan, generating bioactive metabolites such as indole derivatives and indole-3-propionic acid (IPA) [19,20]. These metabolites help maintain intestinal immune homeostasis and protect the epithelial barrier [21,22,23]. Moreover, fermentation derived metabolites may serve as substrates for beneficial gut bacteria such as *Lactobacillus* and *Akkermansia*, supporting their proliferation and contributing to immune regulation [17,24,25]. In the present study, we administered a composite Soybean Fermented Preparation (SFP) to BALB/c mice via oral gavage to evaluate its effects on gut metagenomic composition changes.

This study hypothesized that SFP administration alters gut microbial community composition and is associated with corresponding changes in colonic histological features in BALB/c mice.

## 2. Materials and Methods

### 2.1. Animals

#### 2.1.1. Animals and Experimental Design

Two independent animal experiments were conducted in this study using BALB/c mice (5 weeks old, body weight 18 ± 2 g). First, a sub-chronic toxicity assessment was performed using 32 mice (16 males and 16 females) to evaluate safety across both sexes. Based on the toxicity results, an exploratory gut microbiota and colonic histological analysis was subsequently conducted using a separate cohort of 16 male mice to investigate the effects of SFP on the gut environment.

#### 2.1.2. Animal Housing Conditions

All mice were housed under specific pathogen-free (SPF) conditions with a controlled temperature of 22 ± 2 °C, humidity of 50 ± 10%, and a 12 h light/dark cycle. Standard feed and water were provided ad libitum.

#### 2.1.3. Ethical Statement

All experimental procedures were approved by the Institutional Animal Care and Use Committee of the Catholic University of Pusan (CUP AEC 2023-005).

### 2.2. Components of SFP

The Soybean Fermented Preparation (SFP) used in this study, marketed as Multimune, was supplied by Metagenome Co., Ltd. (Seoul, Republic of Korea). According to the manufacturer, all components of the formulation are produced through a fermentation-based processing method. The formulation consists of 83% soybean fermented powder, 13% fermented kelp (*Laminaria japonica*), and 2% abalone shell (*Haliotis* spp.; Shí Jué Míng), which is processed as part of the fermentation preparation but primarily serves as a mineral-derived component, along with additional fermented extracts of *Astragalus membranaceus*, *Schisandra chinensis*, *Nelumbo nucifera seed*, *Ziziphus jujuba*, and *Artemisia princeps*.

### 2.3. Toxicity Evaluation of SFP

A sub-chronic toxicity study was conducted in accordance with OECD Test Guideline No. 408 (Repeated Dose 90 Day Oral Toxicity Study in Rodents), with minor modifications. A total of 32 BALB/c mice (5 weeks old; 16 males and 16 females; body weight 18 ± 2 g) were randomly assigned into four groups (four males and four females per group). Following a one-week acclimation period, the control group received physiological saline, while the treatment groups administered SFP orally once daily at doses of 500, 1000, or 2000 mg/kg for 60 consecutive days. Throughout the experimental period, all animals were monitored daily for general appearance, behavior, and clinical signs of toxicity.

At the end of each treatment period (60 days), the mice were fasted for 12 h and anesthetized with a combination of alfaxalone (25 mg/kg, Careside, Seongnam, Republic of Korea) and xylazine (5 mg/kg, Bayer Korea, Seoul, Republic of Korea). Blood samples were collected via cardiac puncture; for hematological analysis, blood was drawn into EDTA-coated tubes and analyzed using a DxH 500 Hematology Analyzer (Beckman Coulter, Miami, FL, USA). Plasma obtained after centrifugation at 2500× *g* for 15 min was used for biochemical analysis with an automated biochemistry analyzer (BT1500, Biotecnica Instrument S.p.A., Rome, Italy). Body weight changes were measured every three days using an electronic balance (Ohaus, Parsippany, NJ, USA). After euthanasia, the liver, spleen, kidneys, heart, and lungs were excised, examined for gross abnormalities, and weighed using the same electronic balance to evaluate potential organ-specific toxicity.

### 2.4. Experimental Setup and SFP Treatment in BALB/c Mice

**Figure 1:**
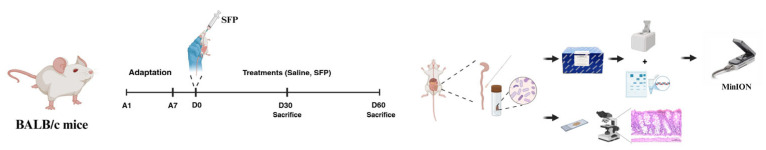
Oral administration schedule and experimental design. The figure was created with BioRender (https://www.biorender.com/, accessed accessed on 2 January 2026).

Male BALB/c mice (5 weeks old at the start of treatment) were acclimated to the experimental environment for one week prior to the initiation of the study. Following acclimation, mice were randomly assigned using a randomized complete block design into four experimental groups as outlined in Table 1. SFP was administered orally once daily at a dose of 1000 mg/kg for either 30 or 60 days, while control groups received vehicle alone. The overall experimental design and treatment schedule are summarized in Figure 1.

This study was designed as an exploratory pilot experiment, and each group consisted of four mice. The limited sample size was selected to generate preliminary descriptive data on microbial and histological changes associated with SFP administration and should be considered when interpreting the results.

### 2.5. Fecal Sample Collection

At the end of each treatment period (30 and 60 days), the mice were anesthetized and euthanized under aseptic conditions. The large intestine, including the cecum and rectum, was carefully dissected, and fecal samples were collected directly from the intestinal lumen using sterile cotton swabs. Each sample was immediately placed into a sterile microtube, rapidly frozen in liquid nitrogen, and stored at −80 °C until analysis.

### 2.6. DNA Extraction and Library Preparation

Genomic DNA was extracted from each fecal sample using the DNeasy PowerSoil Kit (Qiagen, Hilden, Germany) according to the manufacturer’s protocol. DNA concentration and purity were assessed using a NanoDrop spectrophotometer (Thermo Fisher Scientific, Waltham, MA, USA), and DNA integrity was confirmed by electrophoresis on a 1% agarose gel. The full-length rRNA operon region (16S rRNA–ITS–23S rRNA) was amplified using universal primers as described by [26]. Sequencing libraries were prepared using the Ligation Sequencing Kit (Oxford Nanopore Technologies, Oxford, UK) following the manufacturer’s instructions.

Sequencing was performed using the MinION platform (Oxford Nanopore Technologies, Oxford, UK) operated by eGnome Inc. (Seoul, Republic of Korea). Raw nanopore reads were basecalled with Guppy (Oxford Nanopore Technologies), and adapter sequences and low-quality reads were removed using Cutadapt.

The quality-filtered reads were clustered into operational taxonomic units (OTUs) and taxonomically classified using the MIrROR platform (eGnome Inc., Seoul, Republic of Korea), which is based on a curated full-length 16S–23S rRNA operon reference database as previously described [26]. The analytical framework and species-level resolution of operon-based sequencing have been validated in prior studies [27,28].

### 2.7. Microbiota Diversity and Statistical Analyses

Alpha and beta diversity analyses were performed using Python (version 3.11) to evaluate microbial community structure and composition. Alpha diversity indices, including Shannon, Simpson, and Chao1, were calculated to assess microbial richness and evenness. Beta-diversity was determined using the Bray–Curtis dissimilarity index, and the resulting distances were visualized through principal coordinates analysis (PCoA).

### 2.8. Histological Analysis

Colon tissues were collected after euthanasia, fixed in 10% neutral-buffered formalin, and embedded in paraffin. Sections (4 μm) were stained with hematoxylin and eosin (H&E) to assess epithelial integrity, crypt architecture, and overall colonic mucosal morphology. Slides were examined using a DMi1 microscope (Leica, Wetzlar, Germany). Morphometric measurements of mucosal thickness and crypt depth were performed using ImageJ software (version 1.53, NIH, USA). Goblet cell density was quantified as the number of mucin-secreting cells per 100 μm of crypt depth.

### 2.9. Statistical Analysis

All statistical analyses and visualization of microbial composition were conducted using GraphPad Prism software (version 8.4.3, GraphPad Software, San Diego, CA, USA) and Python (version 3.11). Differences between groups were evaluated using one-way analysis of variance (ANOVA) followed by Tukey’s post hoc test, with statistical significance set at *p* < 0.05.

## 3. Results

### 3.1. Sub-Chronic Oral Toxicity of SFP

**Figure 2:**
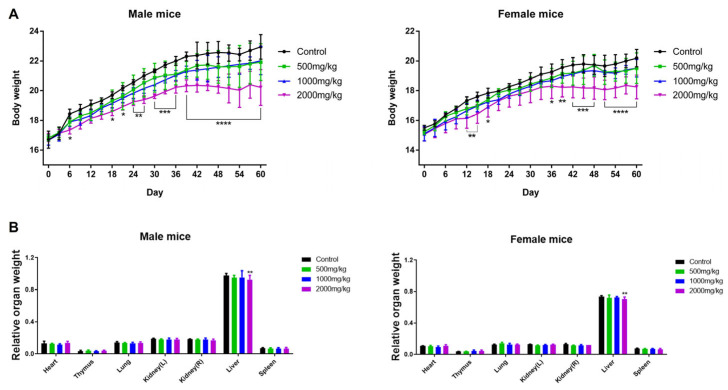
Sub-chronic oral toxicity of SFP in BALB/c mice. Body weight progression (**A**) and relative organ weights (**B**) in male and female BALB/c mice following 60 days of daily SFP administration. Significant weight reduction and decreased liver weight were observed only in the 2000 mg/kg group. Data are presented as mean ± SD (* *p* < 0.05, ** *p* < 0.01, *** *p* < 0.001, **** *p* < 0.0001).

The sub-chronic oral toxicity assessment of SFP was performed in accordance with OECD Test Guideline No. 408 with minor modifications. No mortality or abnormal clinical signs were observed in any treatment group during the 60-day repeated oral administration period. Body weight was monitored every three days, and both male and female BALB/c mice in the 2000 mg/kg group exhibited a significant decrease in body weight compared with controls, whereas no significant changes were noted in the 500 or 1000 mg/kg groups (Figure 2A). At necropsy, major organs were excised and weighed to evaluate potential organ-specific toxicity. Among all examined organs, only liver weight was significantly decreased in both male and female mice administered 2000 mg/kg SFP, while no notable differences were observed in the lower-dose groups (Figure 2B). Collectively, these results indicate that SFP is well tolerated up to 1000 mg/kg, whereas the highest dose of 2000 mg/kg induces mild hepatotoxic changes, reflected by reduced liver weight in the absence of other systemic abnormalities. Complete blood count (CBC) analysis showed no significant changes in any hematological parameters in either male or female mice across all SFP doses (Table A1), indicating that repeated oral administration did not affect hematopoietic function. In contrast, liver function tests (Table A2) revealed dose-dependent hepatic alterations at 2000 mg/kg. Male mice exhibited significant increases in AST, total bilirubin (TB), cholesterol (CHO), and LDH, while female mice showed significant elevations in AST and LDH. No significant biochemical changes were observed at 500 mg/kg or 1000 mg/kg in either sex. Therefore, SFP is considered non-toxic at doses up to 1000 mg/kg, whereas the 2000 mg/kg dose indicates mild hepatotoxicity as evidenced by the elevations in specific hepatic biomarkers.

### 3.2. Gut Microbiota Composition at the Phylum-Level

Across all samples, sequencing generated an average of approximately 63,000 high quality reads per sample (range: 49,267–76,374), with comparable sequencing depth across Control and SFP groups (*n* = 4 per group) at both 30 and 60 days. This sequencing depth was sufficient for robust downstream community-level analyses.

**Figure 3:**
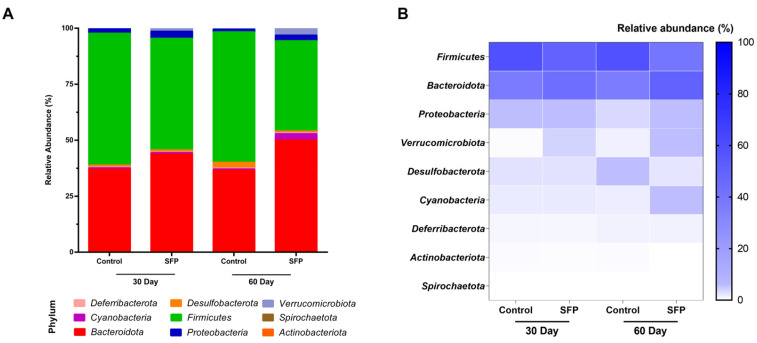
Phylum-level gut microbiota composition and relative abundance patterns of Bacteroidota and Firmicutes following oral administration of SFP (1000 mg/kg/day). (**A**) Stacked bar plots showing the mean relative abundance (%) of bacterial phyla in four experimental groups (Control and SFP at 30 and 60 days). (**B**) Heatmap showing mean relative abundance patterns of dominant and minor phyla across experimental groups.

For further analysis of the overall gut microbial structure, all phyla detected across the dataset were analyzed using fecal samples collected at days 30 and 60. In the updated phylum-level profile (Figure 3A), Bacteroidota and Firmicutes remained the dominant phyla in all groups. On day 30, the SFP-treated group showed a slight increase in Bacteroidota compared to the control group, while Firmicutes constituted the largest proportion in all mice. This pattern shifted by day 60, with Bacteroidota levels (50.05%) exceeding those of the control group (36.5%), establishing a microbial profile in which Bacteroidota became the dominant phylum in SFP-treated mice. Notably, SFP administration also induced distinct changes in less abundant phyla at day 60; specifically, there was a reduction in Desulfobacterota (from 2.58% to 0.80%) and a relative expansion of Cyanobacteria (from 0.55% to 2.83%) and Verrucomicrobiota (from 0.48% to 3.13%) compared to the control group.

The heatmap (Figure 3B) supports these observations, showing consistently stronger Bacteroidota intensity in both the 30-day and 60-day SFP samples. Overall, the updated dataset indicates that SFP supplementation resulted in a reproducible increase in Bacteroidota at both time points, with the most pronounced ecological reorganization including shifts in minor phyla observed at day 60.

### 3.3. Firmicutes/Bacteroidota Ratio and Duncaniella Abundance

**Figure 4:**
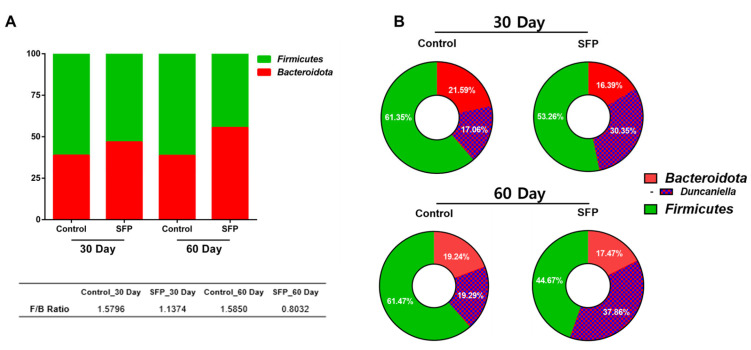
Firmicutes/Bacteroidota (F/B) ratio and relative abundance patterns of *Duncaniella* following oral administration of SFP (1000 mg/kg/day). (**A**) F/B ratio at 30 and 60 days in control and SFP-treated mice. (**B**) Donut charts illustrating relative proportions of Firmicutes, Bacteroidota, and *Duncaniella* at each time point. Data are presented for descriptive comparison.

To further describe phylum and genus-level compositional patterns associated with SFP administration, the Firmicutes/Bacteroidota (F/B) ratio was calculated for each experimental group. As shown in Figure 4A, at day 30, the F/B ratio was 1.5796 in the control group and 1.1374 in the SFP-treated group, indicating a lower ratio in SFP-treated mice. At day 60, the F/B ratio was 1.5850 in control mice and 0.8032 in the SFP-treated group, reflecting a similar pattern of reduced F/B ratio in the presence of SFP.

Within the Bacteroidota phylum, relative abundance patterns at the genus level were further examined, focusing on *Duncaniella* (Figure 4B). At day 30, *Duncaniella* accounted for 17.06% of the microbial community in control mice and 30.35% in SFP-treated mice. At day 60, *Duncaniella* represented 18.29% of the community in control mice and 37.86% in SFP-treated mice. These observations reflect descriptive differences in relative abundance patterns between control and SFP-treated groups at both time points. Due to the limited sample size, no formal statistical testing was applied, and these results should be interpreted as exploratory.

### 3.4. Genus-Level Composition and Differential Abundance

**Figure 5:**
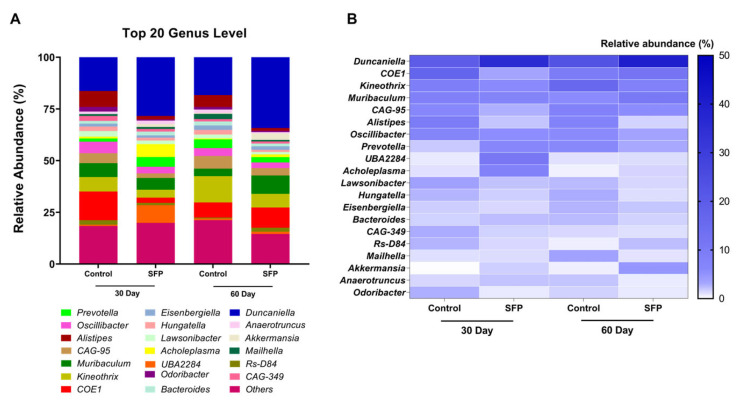
Genus-level composition and relative abundance patterns following oral administration of SFP (1000 mg/kg/day). (**A**) Stacked bar plots showing the mean relative abundance (%) of the top 20 genera in four experimental groups (Control and SFP at 30 and 60 days). (**B**) Heatmap illustrating genus-level relative abundance patterns across experimental groups. Data are presented for descriptive comparison.

Genus-level profiling revealed that the gut microbial community was composed of a shared set of dominant taxa across all experimental groups (Figure 5A). At day 30, control mice showed higher relative abundances of genera such as *Duncaniella*, *COE1*, and *Alistipes*, whereas SFP-treated mice exhibited comparatively higher proportions of *Duncaniella*, *UBA2284*, *Prevotella*, *Acholeplasma*, and *Muribaculum*. At day 60, *Duncaniella* remained a prominent genus in both groups, with *Kineothrix* and *COE1* more apparent in control mice, and *COE1* and *Muribaculum* more apparent in SFP-treated mice. The heatmap visualization (Figure 5B) illustrates these patterns and highlights important temporal shifts in the SFP response. Specifically, the enrichment of *UBA2284*, *Prevotella*, and *Acholeplasma* was a distinct feature of the 30-day SFP-treatment, whereas their proportions decreased by day 60. Conversely, *Muribaculum* and *COE1* showed a more pronounced presence in the SFP group at the 60-day mark compared to their 30-day levels, suggesting a time dependent reorganization of the microbial community.

**Figure 6:**
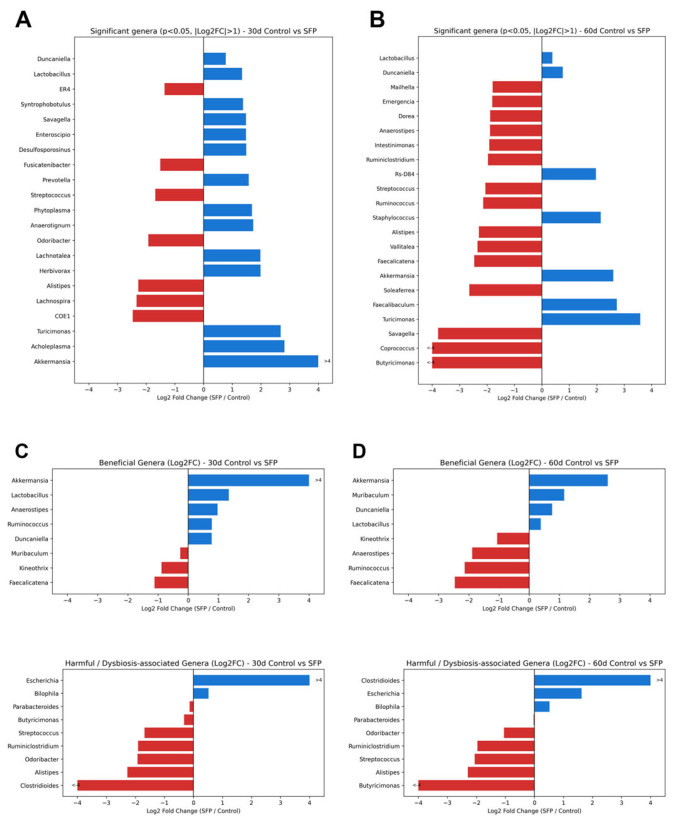
Differential genus abundance analysis between SFP and time-matched control groups. (**A**,**B**) Significant genera (*p* < 0.05, Log_2_ FC > 1) at 30 days (**A**) and 60 days (**B**). Blue bars represent genera enriched in the SFP group, while red bars represent genera enriched in the control group. (**C**,**D**) Log_2_ FC values of health-associated (**C**) and opportunistic genera (**D**), including Escherichia and Bilophila.

While these relative abundance patterns provide a descriptive overview, differential abundance was further quantified using Log_2_ fold change (Log_2_ FC) to identify statistically significant shifts relative to time-matched controls (Figure 6). At day 30, *Akkermansia* (Log_2_ FC > 4), *Acholeplasma*, and *Turicimonas* were significantly enriched in the SFP group (Figure 6A). By day 60, *Turicimonas*, *Faecalibaculum*, and *Akkermansia* maintained robust positive Log_2_ FC values (Figure 6B). In addition to health-associated taxa, the analysis also identified the expansion of opportunistic genera such as *Escherichia* and *Bilophila*, particularly in the 60-day cohort (Figure 6C,D). These complementary analyses demonstrate that SFP administration induces a structured, time-dependent reorganization of the gut microbiota involving both beneficial and opportunistic taxa.

### 3.5. Genus Top 21 Clustered Heatmap

**Figure 7:**
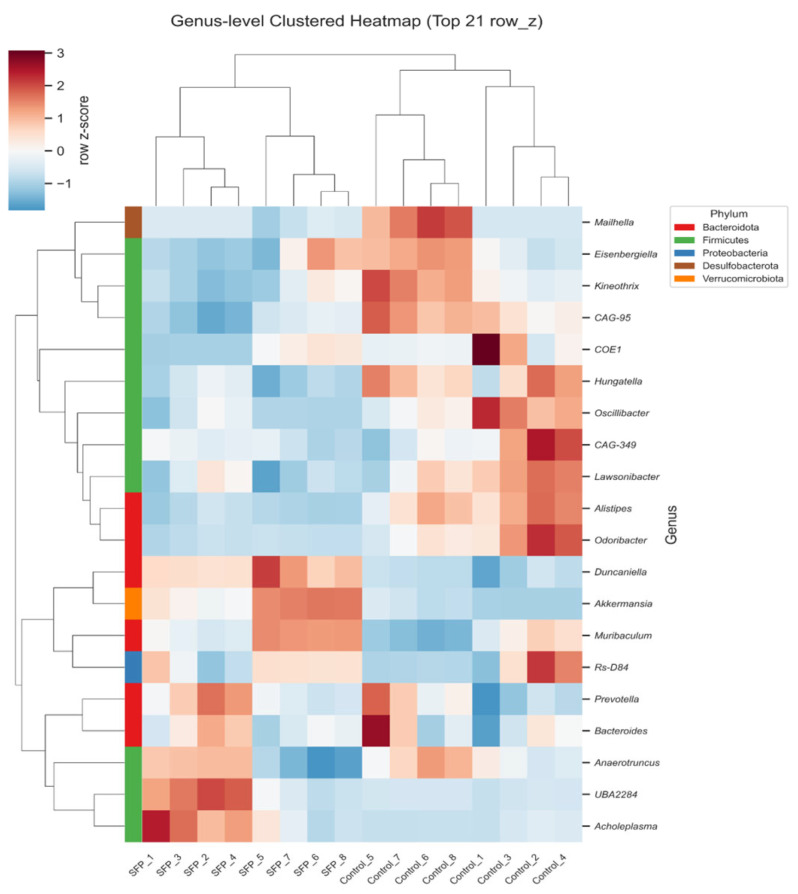
Clustered heatmap of the top 21 most abundant genera across Control and SFP groups. Clustered heatmap showing Z-score normalized genus-level abundance patterns in Control and SFP mice at 30 and 60 days. Hierarchical clustering distinguished SFP-treated and control samples, with clearer separation at 60 days. The heatmap also illustrates temporal shifts in both treatment and control groups, highlighting time specific restructuring of genus-level microbial composition following SFP administration.

Hierarchical clustering of the top genera (Figure 7) showed a clear treatment and time dependent reorganization of the gut microbial community. SFP and control samples formed distinct clusters, with the separation becoming more pronounced at 60 days. Genera enriched in the SFP-treated groups included *Duncaniella*, *Muribaculum*, *Akkermansia*, *UBA2284*, and *Acholeplasma*, forming SFP-associated clusters. Notably, *Prevotella* was also specifically enriched in the 30-day SFP-treated group compared to its corresponding control. Conversely, *Kineothrix*, *Alistipes*, *Oscillibacter*, and *Odoribacter* exhibited higher abundance patterns in the control groups. Furthermore, the baseline microbial composition of the control groups shifted between day 30 and day 60; for instance, genera such as *Kineothrix*, *Eisenbergiella*, and *Mailhella* showed increased abundance in the 60-day control compared to the 30-day control. These reciprocal changes demonstrate that SFP supplementation selectively enhances specific genera belonging to Bacteroidota and Firmicutes, while the overall community also undergoes time dependent shifts in the control groups, contributing to the coordinated restructuring of the gut microbiota.

### 3.6. Genus–Genus Spearman Correlation Coefficient Analysis

**Figure 8:**
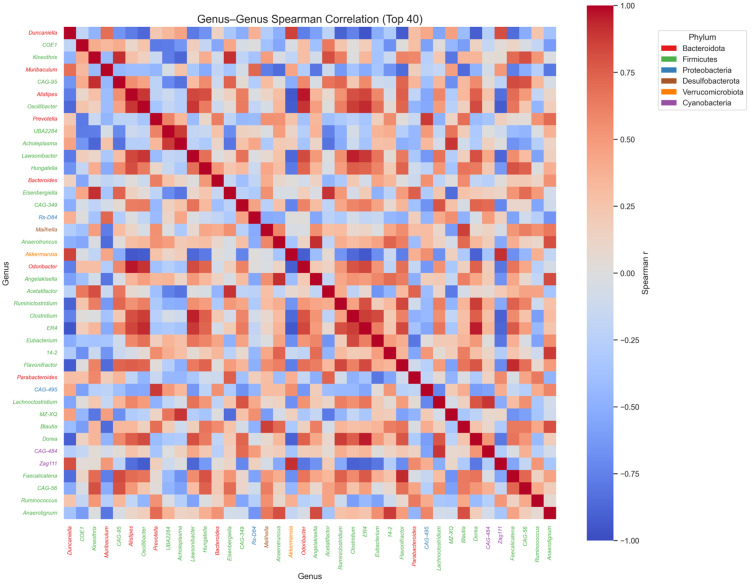
Spearman correlation matrix of the top 40 genera. Colors represent correlation coefficients (ρ), with red indicating positive correlations and blue indicating negative correlations. Genus labels are color-coded by phylum. The matrix highlights distinct co-occurrence and exclusion patterns among taxa. These patterns suggest that SFP-responsive genera tend to cluster together within the correlation structure, while control-dominant taxa form separate ecological groupings.

The Spearman correlation heatmap of the top 40 genera (Figure 8) illustrates the co-occurrence and exclusion patterns that structure the gut microbial community. To ensure an objective and comprehensive analysis, only associations with a Spearman correlation coefficient (r) of 0.6 or higher were considered significant. Under these parameters, *Duncaniella* exhibited strong positive correlations with both *Akkermansia* and *Zag111*, suggesting that these taxa may form a functional guild that responds coordinately to SFP administration. Conversely, several SFP associated genera showed significant negative correlations with tax-prominent control groups. Specifically, *Duncaniella* was negatively associated with *Odoribacter* and *Alistipes*. These results demonstrate that SFP administration induces a structured reorganization of microbial interaction networks, where the expansion of specific SFP responsive genera is systematically linked to the reduction in certain control associated taxa.

### 3.7. Species-Level Composition of the Gut Microbiota

**Figure 9:**
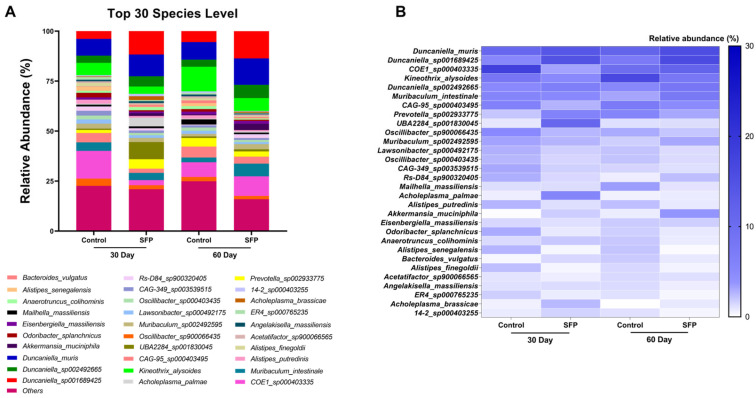
Species-level composition and relative abundance patterns following SFP administration. (**A**) Stacked bar plots showing the mean relative abundance (%) of the top 30 most abundant species across four experimental groups (Control and SFP at 30 and 60 days), ranked by overall relative abundance. (**B**) Heatmap illustrating group-level species abundance patterns across experimental groups and time points, based on relative abundance ranking rather than absolute cut-off values.

Species-level profiling revealed that the gut microbial community was dominated by a consistent set of taxa across all groups, although their relative proportions varied depending on treatment and duration (Figure 9A). Dominant species were identified based on relative abundance ranking within the top 30 most abundant species across groups. In the 30-day Control mice, the most dominant species included *COE1_sp000403335*, *Kineothrix_alysoides*, and *Muribaculum_intestinale*, whereas the 30-day SFP group showed dominance by *Duncaniella_muris*, *Duncaniella_sp001689425*, and *Prevotella_sp002933775*. Notably, *Muribaculum* species also exhibited relatively high abundance in the 30-day SFP group, indicating partial overlap in dominant taxa between groups. At 60 days, the Control group was primarily dominated by *Kineothrix_alysoides*, *Muribaculum_intestinale*, and *COE1_sp000403335*, while the 60-day SFP group displayed a clear dominance of *Duncaniella_muris*, *Duncaniella_sp001689425*, and *Akkermansia_muciniphila*. Although *Duncaniella_muris* remained detectable in Control mice at 60 days, its relative abundance was more pronounced in the SFP-treated group. These changes indicate that although a core set of species structures the community, SFP supplementation shifts their proportional distribution in a time dependent manner. The heatmap (Figure 9B) further highlighted these differences based on grouped analysis rather than individual mice, illustrating distinct species-level abundance patterns between Control and SFP-treated mice at both time points. SFP-treated mice exhibited increased relative abundance of several species, including *Duncaniella_muris*, *Duncaniella_sp001689425*, *Muribaculum_intestinale*, and *Akkermansia_muciniphila*, particularly at 60 days. In contrast, species such as *Odoribacter_splanchnicus*, *Alistipes_putredinis*, and *Anaerotruncus_colihominis* were more abundant in Control groups. Collectively, these findings demonstrate that SFP supplementation is associated with a time dependent redistribution of species-level relative abundance, rather than the emergence of unique taxa. Given the exploratory nature of the study and the limited cohort size, these observations should be interpreted descriptively.

### 3.8. Alpha Diversity Analysis

**Figure 10:**
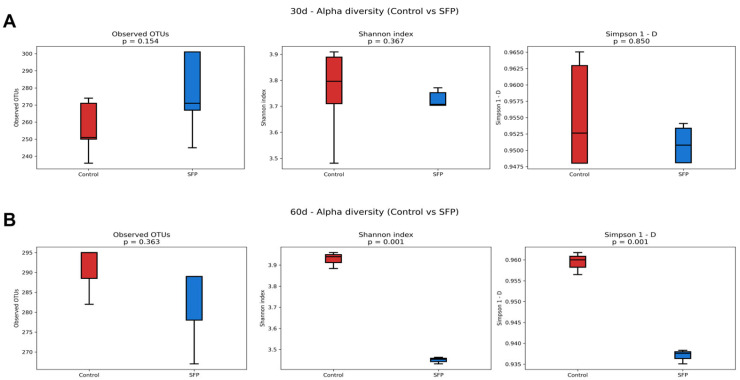
Alpha diversity comparison between Control and SFP groups at 30 and 60 days. (**A**) Alpha diversity indices at 30 days showing no significant differences between Control and SFP groups across all metrics (Observed OTUs, Shannon index, Simpson 1–D). (**B**) Alpha diversity indices at 60 days showing no significant difference in Observed OTUs, while both the Shannon index and Simpson 1–D were significantly reduced in the SFP-treated group, indicating decreased microbial diversity following prolonged SFP administration.

Alpha diversity was evaluated using Observed OTUs, Shannon index, and Simpson index to assess microbial richness and diversity following SFP administration. As shown in Figure 10A, no statistically significant differences were detected between the Control and SFP groups at 30 days across all three indices, indicating that SFP did not alter microbial richness or diversity at the earlier time point. In contrast, at 60 days (Figure 10B), although Observed OTUs remained comparable between groups, both the Shannon (*p* = 0.001) and Simpson (*p* = 0.001) indices were significantly reduced in the SFP-treated mice. These findings indicate a notable decline in microbial diversity rather than richness following prolonged SFP administration. This reduction in diversity indices, despite stable richness, is likely attributed to the increased dominance of specific taxa, such as *Duncaniella*, which may have shifted the microbial community structure toward a less even distribution. Overall, while short-term SFP exposure did not affect alpha diversity, longer-term treatment led to significant decreases in diversity by promoting the dominance of certain microbial groups.

### 3.9. Beta Diversity Analysis

**Figure 11:**
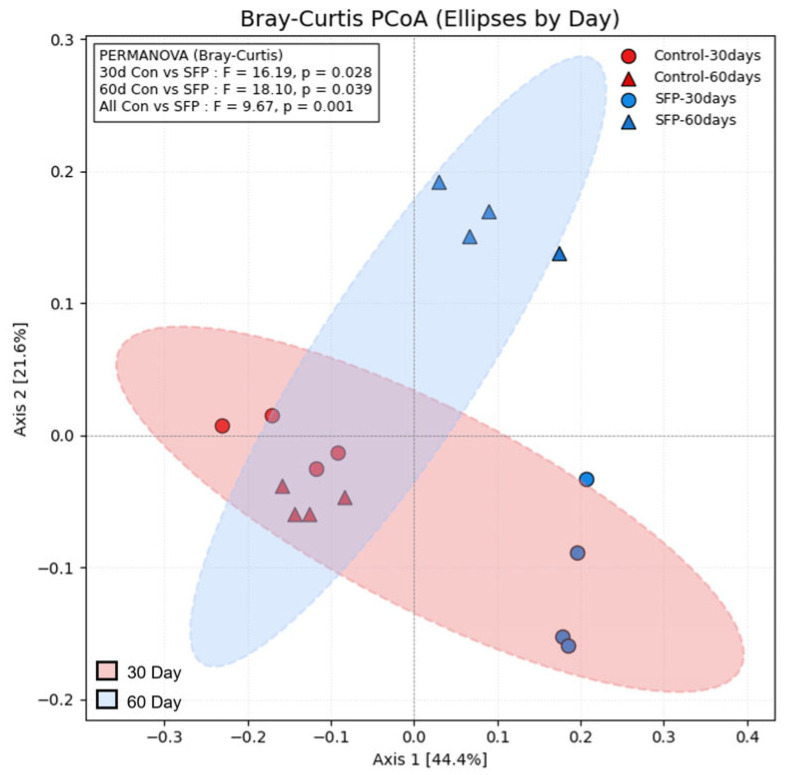
Bray–Curtis PCoA showing β-diversity differences among groups at 30 and 60 days. PCoA plots based on Bray–Curtis distances illustrating β-diversity patterns among Control and SFP groups at 30 and 60 days. Each point represents an individual sample, and ellipses indicate the clustering tendency within each group. PERMANOVA revealed significant differences between Control and SFP groups at both time points (30 days: F = 16.19, *p* = 0.028; 60 days: F = 18.10, *p* = 0.039) and also when all Control samples were compared with all SFP samples (F = 9.67, *p* = 0.001), indicating that SFP supplementation significantly altered overall microbial community structure.

Beta diversity was evaluated using Bray–Curtis distance–based PCoA to compare overall microbial community structure among groups. As shown in Figure 11, PERMANOVA revealed statistically significant differences between the Control and SFP groups at both time points (30 days: F = 16.19, *p* = 0.028; 60 days: F = 18.10, *p* = 0.039), indicating that SFP administration altered the microbial community structure in a time-dependent manner. Furthermore, when all Control samples were compared with all SFP samples regardless of time point, a significant difference was also detected (F = 9.67, *p* = 0.001), supporting a consistent SFP-associated shift in beta diversity across the dataset. The PCoA plot additionally demonstrated clear visual separation between 30-day and 60-day samples, with the 60-day community structures clustering distinctly from the 30-day counterparts. Collectively, these results indicate that SFP supplementation exerted a significant influence on overall microbial community composition, with the effect becoming more pronounced over time.

### 3.10. SFP Increases Goblet Cell Density in the Colonic Mucosa

**Figure 12:**
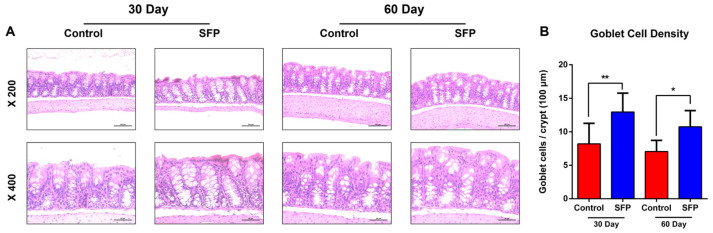
Effects of SFP on colonic mucosal histology and goblet cell density. (**A**) Representative H&E-stained images of distal colon sections from control and SFP-treated mice at day 30 and day 60 (upper: ×200; lower: ×400 magnification). (**B**) Quantification of goblet cell density, expressed as goblet cells per 100 μm of crypt depth. Goblet cell numbers were significantly elevated in the SFP groups compared with controls. Data are presented as mean ± SD. * *p* < 0.05, ** *p* < 0.01 vs. Control.

Representative H&E-stained colonic sections (Figure 12A) showed intact epithelial architecture in both control and SFP-treated mice at 30 and 60 days, with no evidence of overt pathological alterations. Across both time points, SFP-treated mice exhibited a visibly increased number of mucin-producing goblet cells within the colonic crypts compared with controls. Quantitative analysis of goblet cell density, expressed as the number of goblet cells per crypt, demonstrated a significant increase in SFP-treated mice relative to controls at both 30 and 60 days (Figure 12B). These findings indicate that SFP supplementation is associated with measurable alterations in colonic mucosal cellular composition, characterized by increased goblet cell abundance.

## 4. Discussion

Administration of SFP produced a distinct restructuring of the gut microbial community in BALB/c mice, and the nature of these shifts aligns closely with previously reported microbiota immune interactions. *Akkermansia* has similarly been highlighted for its ability to maintain epithelial barrier integrity and augment responsiveness to immune checkpoint inhibitors, while Lactobacillus contributes to mucosal repair and immunomodulation [29,30,31,32,33]. These collective findings indicate that enrichment of these taxa may confer systemic benefits extending beyond gut homeostasis.

In this study, the most prominent feature of the microbial restructuring induced by SFP supplementation was the marked increase in *Duncaniella*, which became one of the dominant genera in SFP treated groups. This pattern is consistent with prior evidence showing that *Muribaculaceae* benefit from dietary substrates associated with fermentation and contribute to intestinal metabolic regulation [34,35]. Previous studies on soybean fermentation have reported similar shifts in gut microbial composition, including enrichment of fermentative and polysaccharide-utilizing taxa. For example, fermented soybean products have been shown to modulate gut microbiota structure and promote the expansion of metabolically active bacterial groups, supporting the notion that soybean-derived fermentation substrates can drive reproducible microbial remodeling [36].

Importantly, SFP lowered the Firmicutes/Bacteroidota (F/B) ratio. While the F/B ratio is often discussed as a marker of metabolic health, its interpretation remains subject to context dependency, as shifts in this ratio do not universally correlate with health outcomes across different dietary models. In this study, however, the reduced ratio, driven largely by the expansion of *Duncaniella*, suggests a reorganization toward a profile often observed in metabolic intervention studies [37,38].

Similarly, *Akkermansia* and *Lactobacillus*, two well established taxa linked to epithelial barrier protection and immune regulation, were significantly elevated in SFP treated animals, and a strong positive correlation between *Duncaniella* and *Akkermansia* indicated a coordinated shift toward health-associated microbial networks [7,11,31,39].

In contrast, genera previously reported to be associated with dysbiotic and inflammatory gut conditions, including *Odoribacter* and *Prevotella*, were reduced following SFP administration, and these changes were consistently reflected across compositional analyses and correlation matrices, where *Duncaniella* showed strong negative associations with these taxa [40,41].

SFP supplementation maintained overall richness, as evidenced by stable Observed OTUs at 60 days; however, it significantly altered community evenness, leading to decreased Shannon and Simpson indices. The observed reduction in these diversity indices indicates a shift in microbial distribution rather than a loss of taxa. This phenomenon is primarily attributed to the selective dominance of specific taxa, such as *Duncaniella*, which expanded at the expense of community evenness. While a decrease in evenness can sometimes be interpreted as a sign of microbial instability, in the context of SFP administration, it more likely reflects a time dependent selective enrichment of specialized fermentative taxa.

Notably, the extent of this shift differed between the 30- and 60-day cohorts. Microbial restructuring was already evident at 30 days, whereas prolonged SFP administration for 60 days resulted in a more pronounced dominance pattern. This temporal divergence suggests that SFP induced modulation of the gut microbiota progresses from an early adaptive response toward a more stabilized, selectively enriched community structure with continued exposure. This pattern suggests not a loss of taxa, but a selective expansion of specific microbial groups. β-diversity analysis further demonstrated clear separation between SFP-treated and control mice at both time points, confirmed by PERMANOVA, indicating that SFP induced a time-dependent reorganization of microbial community structure [2,37].

The SFP induced enrichment of several dominant taxa also expanded species-level compositional complexity at deeper taxonomic levels, as species-level profiling revealed expanded populations of *Duncaniella_muris*, *Duncaniella_sp001689425*, *Muribaculum_intestinale*, and *Akkermansia_muciniphila*. These shifts imply that SFP not only affects higher-level genera but also promotes diversification within functionally relevant species groups. Such expansion of species level representation is advantageous because a more taxonomically diverse gut microbiota tends to exhibit greater metabolic redundancy, resilience to perturbation, and enhanced capacity to maintain epithelial barrier stability [10,34,35].

Histological evaluation demonstrated a significant increase in goblet cell numbers in SFP-treated mice. While goblet cells play a key role in mucin production and mucus layer maintenance, the current study was not designed to directly assess epithelial barrier function or inflammatory status. Therefore, the observed increase in goblet cell density should be interpreted descriptively rather than as definitive evidence of improved epithelial defense. Notably, increased mucus production can reflect multiple biological contexts, including adaptive or compensatory responses to microbial or dietary stimuli. In particular, the expansion of mucus-associated taxa such as *Akkermansia muciniphila*, especially following prolonged SFP administration, may contribute to increased mucin turnover.

This process could potentially lead to compensatory mucus production rather than unequivocal barrier enhancement. Accordingly, while the histological findings indicate that SFP administration is associated with measurable changes in colonic mucosal features, further functional studies are required to determine whether these alterations represent beneficial reinforcement of epithelial defense or adaptive responses to sustained microbial remodeling [31,32,33,42].

In summary, these results indicate that SFP supplementation induces a favorable remodeling of the gut microbiota characterized by enrichment of immunoregulatory and mucosa supporting taxa, reduction in dysbiosis associated genera, normalization of the F/B ratio, and enhancement of species-level compositional breadth. When interpreted alongside prior literature, the microbial alterations observed in this study suggest that SFP promotes a health-associated ecological shift with the potential to contribute to improved mucosal stability and immune resilience, providing a mechanistic foundation for understanding the broader benefits of fermented food derived complexes on host physiology [2,15,31].

From a translational perspective, the ability of SFP to modulate key microbial taxa without inducing systemic toxicity as observed in our safety assessment suggests its potential as a functional dietary ingredient. However, the practical application of these findings requires a cautious approach. The reproducibility of these microbial shifts in broader contexts may be influenced by baseline microbiota diversity and individual metabolic variability. Furthermore, while the current dose provided clear ecological changes in a controlled model, further research is necessary to determine the optimal intake levels and long-term stability required for consistent efficacy in diverse populations.

Despite these promising findings, several limitations should be considered when interpreting the results of this study. One important limitation is that the cohort size was relatively limited, which may constrain statistical power and the generalizability of the observed microbial and histological changes. In addition, the SFP used in this study was administered as a commercially available multicomponent preparation (Multimune), which contains additional fermented plant and marine derived constituents. Therefore, the observed effects cannot be attributed exclusively to soybean fermentation alone, and future studies incorporating refined or component specific controls will be necessary to delineate the contribution of individual constituents. The metagenomic analysis was restricted to taxonomic profiling without parallel functional or metabolomic assessments, limiting mechanistic interpretation. Moreover, direct host–microbe interaction pathways were not evaluated, necessitating further studies to elucidate whether SFP-induced microbial remodeling translates into measurable immune or mucosal outcomes.

These observations differ in part from a previous study reporting that administration of a soybean fermented preparation increased bacterial diversity and was accompanied by weight gain following a relatively short treatment period [43]. Several factors may account for these differences. Notably, the duration of SFP exposure differed substantially between the two studies, as the present work focused on prolonged administration (30 and 60 days), whereas the previous study evaluated a shorter intervention period.

In this context, increased microbial diversity observed during short-term supplementation may reflect an early and transient expansion of multiple taxa in response to novel dietary substrates. In contrast, the reduced Shannon and Simpson indices observed at 60 days in the present study indicate a shift in community evenness driven by selective dominance of specific taxa rather than a loss of microbial richness. Such time-dependent ecological restructuring has been described in other dietary intervention studies and may reflect a progression from initial diversification toward a more stabilized microbial configuration.

Importantly, increased bacterial diversity does not uniformly translate into favorable metabolic outcomes, as demonstrated by the weight gain reported in the previous study [43]. Although metabolic parameters were not assessed in the current study, these contrasting findings highlight the importance of treatment duration and host context when interpreting diversity metrics. Further studies integrating metabolic and functional analyses will be required to clarify the physiological implications of prolonged SFP-induced microbial dominance.

## 5. Conclusions

Long-term supplementation with Soybean Fermented Preparation (SFP) induced distinct and consistent changes in the gut microbial community of BALB/c mice. SFP intake resulted in clear β-diversity separation from controls at both 30 and 60 days, indicating a persistent restructuring of microbial composition. Notably, SFP supplementation enriched beneficial taxa, including *Akkermansia*, *Lactobacillus*, and members of the *Muribaculaceae* family such as *Duncaniella*, which are known to be associated with mucosal protection and metabolic regulation. The reduction in several dysbiosis-associated genera further suggests that SFP helps shift the microbiota toward a more favorable ecological state. Histological evaluation demonstrated that these microbial alterations were accompanied by improvements in colonic mucosal structure. SFP-treated mice exhibited comparable epithelial architecture to controls, along with a significant increase in goblet cell density. Together, these findings indicate that SFP supplementation is associated with measurable histological changes in the colonic mucosa and concurrent alterations in gut microbial composition.

While this study identifies key microbial and histological outcomes associated with long-term SFP intake, functional mechanistic analyses such as metabolomic profiling or integrative pathway evaluation will be required in future research to clarify how specific microbial shifts contribute to host physiological benefits.

## Figures and Tables

**Table 1:** Experimental Group Design.

Group No.	Designation	Duration (Day)	Treatment	*n*
Group 1	Control	30	Saline	4
Group 2	SFP		SFP 1000 mg/kg	4
Group 3	Control	60	Saline	4
Group 4	SFP		SFP 1000 mg/kg	4

**Figure A1:**
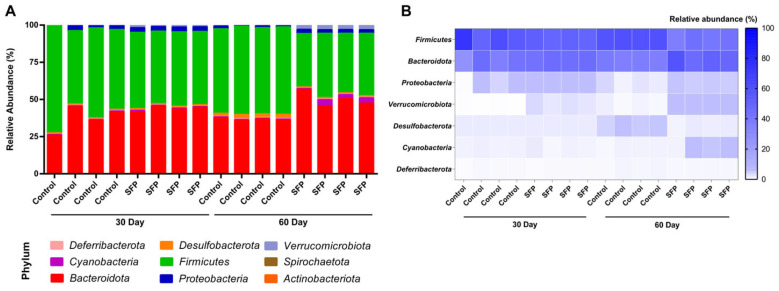
Phylum-level gut microbiota composition in BALB/c mice following SFP administration at 30 and 60 days. (**A**) Stacked bar plots showing the relative abundance of all detected phyla at 30 and 60 days. Bacteroidota and Firmicutes were dominant in all groups, with Bacteroidota increasing modestly at 30 days and more clearly at 60 days in SFP-treated mice. (**B**) Heatmap illustrating the phylum-level abundance patterns across groups. Higher Bacteroidota and lower Firmicutes are observed in SFP-treated mice, particularly at 60 days. Color intensity indicates relative abundance.

**Figure A2:**
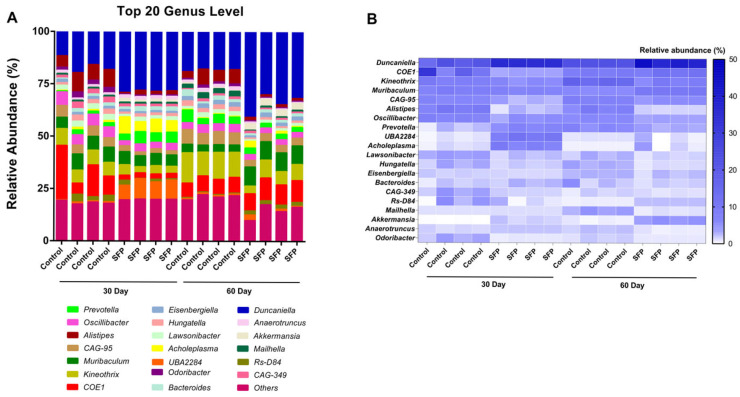
Genus-level composition and relative abundance patterns following SFP administration. (**A**) Stacked bar plots showing the top 20 genera in control and SFP groups at 30 and 60 days. (**B**) Heatmap depicting genus-level abundance patterns. SFP-treated mice exhibited increased levels of *Duncaniella*, *Muribaculum*, *Akkermansia*, *UBA2284*, and *COE1*, while *Kineothrix*, *Alistipes*, and *Oscillibacter* were more abundant in the control groups. These shifts illustrate selective enrichment of Bacteroidota and Firmicutes associated genera following SFP intake.

**Figure A3:**
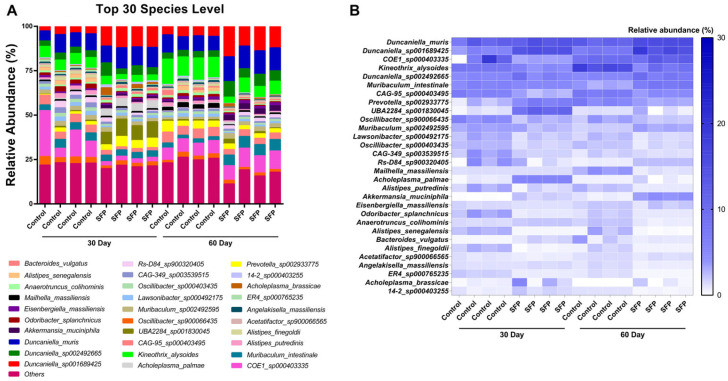
Species-level composition and relative abundance patterns following SFP administration. (**A**) Stacked bar plots showing the top 30 most abundant species in control and SFP groups at 30 and 60 days. (**B**) Heatmap illustrating species-level abundance changes, highlighting increased levels of *Duncaniella*, *Muribaculum*_*intestinale*, and *Akkermansia*_*muciniphila* in the SFP treated mice, especially at 60 days.

## Data Availability

The original contributions presented in this study are included in the article. Further inquiries can be directed to the corresponding authors.

## Appendix A

**Table A1:** Hematological profiles of BALB/c mice following 60 days of oral SFP administration.

Parameters	Control	SFP (mg/kg)		
		500	1000	2000
Male				
WBC (x10^3^/uL)	1.420 ± 0.156	1.037 ± 0.350	1.028 ± 0.291	1.155 ± 0.465
LY (%)	90.867 ± 3.276	91.867 ± 4.290	95.200 ± 1.797	90.518 ± 3.450
MO (%)	2.507 ± 0.203	2.137 ± 0.371	0.750 ± 0.395	1.090 ± 0.770
NE (%)	4.927 ± 3.262	6.047 ± 3.568	3.318 ± 1.333	7.740 ± 2.266
EO (%)	0.513 ± 0.078	0.447 ± 0.164	0.355 ± 0.182	0.470 ± 0.094
BA (%)	0.343 ± 0.125	0.167 ± 0.078	0.378 ± 0.170	0.183 ± 0.102
RBC (×10^6^/uL)	8.297 ± 0.652	8.790 ± 0.227	8.650 ± 0.160	8.980 ± 0.103
PLT (×10^3^/uL)	385.167 ± 61.267	413.767 ± 33.379	382.425 ± 36.237	405.750 ± 93.62
HGB (g/dL)	14.320 ± 0.439	15.007 ± 0.358	14.900 ± 0.469	15.490 ± 0.163
HCT (%)	43.767 ± 3.229	45.600 ± 1.275	45.450 ± 1.607	47.375 ± 0.402
MCV (fL)	52.767 ± 0.403	51.900 ± 0.141	52.550 ± 1.453	52.750 ± 0.512
MCH (pg)	17.333 ± 0.929	17.033 ± 0.094	17.225 ± 0.426	17.225 ± 0.043
MCHC (g/dL)	32.833 ± 1.558	32.933 ± 0.249	32.825 ± 0.618	32.675 ± 0.356
Parameters	Control	SFP (mg/kg)		
		500	1000	2000
Female				
WBC (×10^3^/uL)	1.057 ± 0.186	0.667 ± 0.219	0.583 ± 0.413	0.483 ± 0.254
LY (%)	90.873 ± 1.090	93.487 ± 2.275	89.728 ± 5.113	91.975 ± 2.288
MO (%)	2.077 ± 1.389	1.12 ± 0.652	1.365 ± 0.841	0.888 ± 1.157
NE (%)	5.913 ± 2.213	4.647 ± 1.643	7.078 ± 4.229	5.718 ± 2.137
EO (%)	0.587 ± 0.392	0.383 ± 0.193	1.553 ± 1.193	0.993 ± 0.415
BA (%)	0.550 ± 0.481	0.363 ± 0.190	0.280 ± 0.092	0.428 ± 0.217
RBC (×10^6^/uL)	9.063 ± 0.256	9.060 ± 0.401	9.300 ± 0.273	9.073 ± 0.362
PLT (×10^3^/uL)	372.233 ± 73.796	397.633 ± 56.884	404.8 ± 105.231	412.25 ± 171.575
HGB (g/dL)	15.627 ± 0.221	15.670 ± 0.462	15.713 ± 0.330	15.210 ± 0.517
HCT (%)	47.333 ± 1.541	47.433 ± 1.429	47.775 ± 1.619	46.000 ± 1.909
MCV (fL)	52.267 ± 0.236	52.367 ± 0.741	51.350 ± 0.350	50.700 ± 0.552
MCH (pg)	17.267 ± 0.262	17.300 ± 0.294	16.900 ± 0.224	16.775 ± 0.148
MCHC (g/dL)	33.033 ± 0.602	33.033 ± 0.170	32.900 ± 0.678	33.075 ± 0.476

**Table A2:** Serum biochemical and liver function test results following 60 days of SFP administration.

Parameters	Control	SFP (mg/kg)		
		500	1000	2000
Male				
ALP (U/L)	16.533 ± 1.399	17.109 ± 6.071	23.068 ± 12.214	17.498 ± 4.686
ALT (U/L)	22.000 ± 3.559	22.400 ± 1.883	23.600 ± 2.466	40.234 ± 17.637
AST (U/L)	86.417 ± 8.643	77.787 ± 14.489	109.960 ± 26.775	121.043 ± 26.312 **
TB (mg/dL)	0.537 ± 0.027	0.584 ± 0.039	0.627 ± 0.047	1.126 ± 0.298 *
TP (g/dL)	4.328 ± 0.149	4.537 ± 0.200	4.828 ± 0.242	5.762 ± 1.204
TG (mg/dL)	38.233 ± 5.711	31.707 ± 4.867	42.615 ± 7.377	43.133 ± 10.179
ALB (g/dL)	2.325 ± 0.195	2.638 ± 0.120	2.768 ± 0.106	3.255 ± 0.725
CHO (mg/dL)	84.983 ± 7.668	94.233 ± 8.521	96.640 ± 4.631	117.941 ± 25.131 *
LDH (U/L)	253.667 ± 44.535	243.867 ± 51.107	270.933 ± 16.993	291.727 ± 69.555 **
HDL (mg/dL)	77.283 ± 6.465	81.267 ± 1.901	86.620 ± 4.438	93.298 ± 11.150
Parameters	Control	SFP (mg/kg)		
		500	1000	2000
Female				
ALP (U/L)	18.434 ± 6.396	15.240 ± 1.815	26.096 ± 15.886	23.394 ± 16.851
ALT (U/L)	25.300 ± 4.901	20.700 ± 1.364	35.325 ± 3.462	34.163 ± 16.380
AST (U/L)	142.097 ± 28.974	123.993 ± 35.757	158.575 ± 25.657	171.666 ± 29.13 *
TB (mg/dL)	0.693 ± 0.071	0.679 ± 0.083	0.721 ± 0.084	0.896 ± 0.199
TP (g/dL)	4.611 ± 0.103	4.633 ± 0.144	4.808 ± 0.138	4.727 ± 0.102
TG (mg/dL)	50.020 ± 3.247	35.220 ± 7.866	34.615 ± 2.185	40.271 ± 9.474
ALB (g/dL)	2.704 ± 0.121	2.730 ± 0.127	3.006 ± 0.121	2.782 ± 0.164
CHO (mg/dL)	66.670 ± 3.482	74.510 ± 12.473	61.080 ± 4.558	68.333 ± 0.651
LDH (U/L)	268.733 ± 40.684	251.067 ± 30.715	287.875 ± 42.508	303.288 ± 57.063 **
HDL (mg/dL)	59.010 ± 2.701	65.230 ± 7.152	50.070 ± 2.230	59.261 ± 2.129

Liver function tests (LFT); * *p* < 0.05; ** *p* < 0.01 VS Control.
